# Adolescents and primary care

**DOI:** 10.1186/1824-7288-41-S2-A57

**Published:** 2015-09-30

**Authors:** Marina Picca, Roberto Marinello

**Affiliations:** 1 SICuPP, Milan, Italy

## 

From 1978 National Health System (NHS) primary care pediatricians (PdF) in Italy take care of infants and adolescents from birth until 14 (in selected cases 16) years of age [1]. They are not allowed to take care of their patients up to 18 years, so it's not possible to follow the whole adolescence under the NHS. There is also increased awareness in families that the pediatrician is the natural medical reference of a teenager; their education and training allows the understanding of changes due to puberty and their experience and background permit thorough understanding not only of the physical but also of the psychological and social changes which belong to this period of transition [2]. However, pediatricians are increasing their knowledge and competence regarding adolescent care and are promoting new preventive programs for adolescent health, such as the “Adolescents Health Project” to strengthen the preventative and educative actions and improve the lifestyle of the young.

Another objective of primary care pediatricians will be to structure shared programs with general practitioners (MMG) to favour the shift of care from the primary care pediatrician to the family doctor. It should be necessary to always support a well planned care transition between Pdf and MMG (exchange of computerized or paper medical records and when possible direct contact). This will enable the MMG to receive the complete information on the health of the patient as well as on social and relational aspects.
